# Adjuvant Hysterectomy for Cervical Cancer Patients Treated with Chemoradiation Therapy: A Systematic Review on the Pathology-Proven Residual Disease Rate

**DOI:** 10.3390/cancers13246190

**Published:** 2021-12-08

**Authors:** Kim van Kol, Renée Ebisch, Jurgen Piek, Maaike Beugeling, Tineke Vergeldt, Ruud Bekkers

**Affiliations:** 1 Department of Obstetrics and Gynecology and Catharina Cancer Institute, Catharina Hospital, 5623 EJ Eindhoven, The Netherlands; k.vankol@student.maastrichtuniversity.nl (K.v.K.); renee.ebisch@radboudumc.nl (R.E.); 2 Department of Obstetrics and Gynecology, GROW School for Oncology and Developmental Biology, Maastricht University Medical Center+, 6229 ER Maastricht, The Netherlands; 3 Department of Obstetrics and Gynecology, Radboud University Medical Center, 6525 XZ Nijmegen, The Netherlands; jurgen.piek@catharinaziekenhuis.nl (J.P.); tineke.vergeldt@radboudumc.nl (T.V.); 4 Department of Radiation Oncology, Institute Verbeeten (BVI), 5042 SB Tilburg, The Netherlands; beugeling.m@bvi.nl

**Keywords:** locally advanced cervical cancer, chemoradiation therapy, adjuvant hysterectomy, complications, survival

## Abstract

**Simple Summary:**

The treatment for patients with locally advanced cervical cancer generally consists of platinum-based chemotherapy during external beam radiotherapy, followed by brachytherapy. Some medical centers perform adjuvant hysterectomy after chemoradiation therapy, even though the international guideline advises otherwise. Performing adjuvant hysterectomy after chemoradiation therapy is associated with a high complication rate and the percentage residual disease in adjuvant hysterectomy specimen is unknown. Therefore, the aim of our systematic review was to determine the percentage of residual disease in the adjuvant hysterectomy specimen. Furthermore, we want to determine if there is an association between the time of adjuvant hysterectomy and the percentage residual disease in adjuvant hysterectomy specimens. Findings from this research provide insight into potential complications, survival benefits, and an overtreatment rate. Patients need to be well informed before considering an adjuvant hysterectomy.

**Abstract:**

Objective: To determine the incidence of pathology-proven residual disease in adjuvant hysterectomy specimens in patients with cervical cancer, treated with chemoradiation therapy. Secondly, to assess a possible association for pathology-proven residual disease regarding the time between chemoradiation therapy and adjuvant hysterectomy. Additionally, the survival rate and complication rate were assessed. Methods: PubMed, EMBASE, and the Cochrane database were searched from inception up to 8 March 2021. Results: Of the 4601 screened articles, eleven studies were included. A total of 1205 patients were treated with chemoradiation therapy and adjuvant hysterectomy, ranging from three to twelve weeks after chemoradiation therapy. A total of 411 out of 1205 patients (34%) had pathology-proven residual disease in the adjuvant hysterectomy specimen. There was no association found in the time between chemoradiation therapy and adjuvant hysterectomy. Follow-up ranged from 2.4 to 245 months, during which 270 patients (22%) relapsed, and 298 patients (27%) were deceased. A total of 202 (35%) complications were registered in 578 patients. Conclusion: there is no association found in the time between chemoradiation therapy and residual disease on adjuvant hysterectomy specimens. The survival rates after chemoradiation therapy and adjuvant hysterectomy are suboptimal, while the risk of complications after adjuvant hysterectomy is high.

## 1. Introduction

Cervical cancer is the fourth most common malignancy in patients worldwide, within 2018, an estimated number of 570,000 patients were diagnosed with cervical cancer [1,2]. Approximately 30–40% of patients with cervical cancer in developed countries have locally advanced cervical cancer at initial diagnosis [3]. Locally advanced cervical cancer is classified as stage IIB-IVA according to the International Federation of Gynecology and Obstetrics (FIGO) 2018 staging system [2]. 

According to international guidelines, the standard treatment for patients with locally advanced cervical cancer is concurrent chemoradiation therapy. This treatment generally consists of platinum-based chemotherapy during external beam radiotherapy, followed by brachytherapy [4]. There is still a reported overall local pelvic recurrence rate of 14.5% after treatment with chemoradiation therapy for patients with stage IIB-IVA disease [5,6]. Reported 5-year overall survival rates range from 83% for stage IB to 32% for stage IVA [7]. Even though the international guidelines advise otherwise, some medical centers do perform adjuvant hysterectomy after chemoradiation therapy. Adjuvant hysterectomy after chemoradiation therapy has not been shown to be effective in improving survival outcomes, but it seems to reduce the risk of local recurrence [8,9].

If a patient has received chemoradiation therapy, the morphologic response continues after chemoradiation therapy. Therefore, the total benefit of chemoradiation therapy is achieved for several weeks after the last radiation treatment [10]. Medical centers performing routine adjuvant hysterectomy are inconsistent in the time between chemoradiation therapy and adjuvant surgery. However, the time between chemoradiation therapy and adjuvant hysterectomy has not been assessed before [4,9]. Performing adjuvant hysterectomy after chemoradiation therapy is associated with a high complication risk because of radiation-induced tissue damage and reduced propensity to healing [11,12]. This may negatively influence the quality of life of patients. 

The aim of this review is to determine the incidence of pathology-proven residual disease in adjuvant hysterectomy specimens in patients treated with chemoradiation therapy because of cervical cancer. Furthermore, we aimed to assess a possible association with time between chemoradiation therapy and adjuvant hysterectomy. Secondary outcome measures were survival rate and complications after an adjuvant hysterectomy.

## 2. Materials and Methods

### 2.1. Protocol and Registration

A protocol was designed according to the Preferred Reporting Items for Systematic Reviews and Meta-Analyses (PRISMA) guideline and registered in PROSPERO (registration number CRD42020196399). In this systematic review, adjuvant hysterectomy was defined as planned surgery after primary treatment with chemoradiation therapy, without determining the presence or absence of residual disease. Adequate chemoradiation therapy is defined as a total dose radiation therapy of at least 70 Gy (external beam radiation therapy and brachytherapy). Preferable, with daily external beam radiotherapy total dose of 45 Gy with concomitant platinum-based chemotherapy (recommended weekly cisplatin 40 mg/m^2^ for 5–6 cycles) followed by brachytherapy.

### 2.2. Literature Search

PubMed, EMBASE and Cochrane library were searched for articles published from inception up to 8 March 2021. The selection criteria combined synonyms for cervix uteri, cervical cancer, chemoradiation therapy, hysterectomy and adjuvant hysterectomy and included MeSH terms (Supporting Information S1). Duplicate articles were manually filtered using the bibliographic database Endnote X9. 

### 2.3. Eligibility Criteria

All titles and abstracts were independently assessed by two researchers (KvK, RE) and any discrepancies were resolved by a third researcher (TV). The selection of articles was independently assessed for full text by two reviewers. Studies describing patients with cervical cancer, requiring chemoradiation therapy, and treated with adjuvant hysterectomy were suitable for inclusion. In this systematic review, there were no restrictions regarding language and the different FIGO-classifications. The time between chemoradiation therapy and adjuvant hysterectomy, the pathology results after adjuvant hysterectomy, and survival rates had to be described in the study. Reviews of literature, case reports and case series with five patients or less, conference abstracts and letters to the editor were excluded. Articles describing patients treated with salvage hysterectomy and studies treating patients with inadequate chemoradiation therapy were not included. Studies were included when >90% of patients were treated according to our adequate chemoradiation therapy definition. For studies updating prior published series, the most recent data were retained. To reduce the risk of including duplicate patients in the systematic review, articles written by the same authors and/or university were assessed by three reviewers (KvK, RE, TV), based on year, inclusion period, number of patients, disease stage, chemoradiation therapy regimen, surgical treatment, and the follow-up period. From coinciding articles, the articles with the most recent information, the most included patients, the broadest stage range, and the largest follow-up period were included. 

### 2.4. Data Collection

From the relevant articles, the following information was extracted: inclusion period, country, number of patients treated with chemoradiation therapy, number of patients with adjuvant hysterectomy, FIGO stage, histology, dose and regimen of chemoradiation therapy, dose of brachytherapy, type of adjuvant hysterectomy, time until adjuvant hysterectomy, pathology results after surgery, complications after adjuvant hysterectomy, recurrence rate and survival outcome. An association between CRT and adjuvant hysterectomy was considered when the percentage of positive pathology diminished in time. The overall survival rate was calculated by dividing the number of deaths by the total of people treated with chemoradiation therapy and adjuvant hysterectomy. The complication rate was calculated by dividing the number of reported complications by the number of patients treated with chemoradiation therapy and adjuvant hysterectomy.

### 2.5. Assessment of Risk of Bias

Two reviewers (KvK, RE) independently determined the quality of the included articles according to the Newcastle-Ottawa Scale for cohort studies. The quality assessment was based on three categories: selection, comparability, and outcomes. A total of nine stars could be awarded, a study with six or more stars was defined as a high-quality study, between three and five stars was defined as fair quality and less than three stars as poor quality. 

## 3. Results

**Figure 1:**
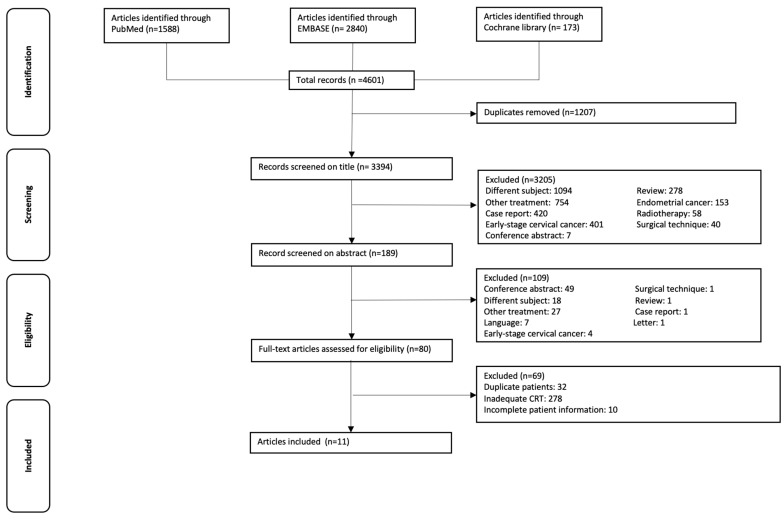
Flowchart of the selection procedure.

The search revealed 4601 articles after systematical removal of duplicates. After screening title and abstract, 80 studies were evaluated for full text. After reading full text, 69 studies did not fulfill the search query, 32 studies were excluded based on the same cohorts of patients, and 27 studies were excluded because of inadequate chemoradiation therapy. Ten studies were excluded because of incomplete patient information (time till adjuvant hysterectomy, pathology results, follow-up, recurrence, or survival rate were missing). No additional studies were identified by checking the reference list. Figure 1 shows the flow diagram of the selection process.

### 3.1. Characteristics of Included Studies

The total of eleven included articles consisted of six retrospective cohort studies, four prospective cohort studies and one phase III randomized controlled trial. Results of the included studies [13,14,15,16,17,18,19,20,21,22,23] are summarized in Table 1. 

### 3.2. Newcastle-Ottawa Scale

Of the total group of eleven studies, ten studies were assessed as good or fair quality and one study was assessed as poor quality according to the Newcastle-Ottawa Scale for cohort studies (Table 2). 

### 3.3. Analysis

**Figure 2:**
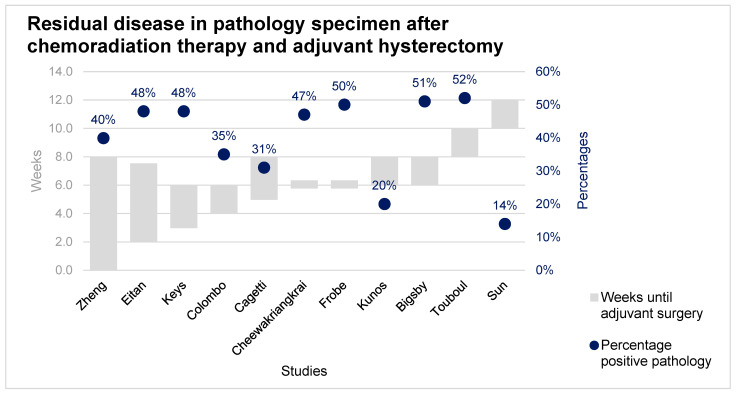
Percentage pathology-proven residual disease after adequate chemoradiation therapy and adjuvant hysterectomy.

Eleven studies were included, representing a total of 2077 patients of which 1205 patients (58%) were treated with chemoradiation therapy and adjuvant hysterectomy. Patients were included with an age range of 19–90 years. Of this group, 955 (79%) patients were diagnosed with squamous cell carcinoma, 158 (13%) with adenocarcinoma, 37 (3%) with adenosquamous carcinoma and 55 (5%) with another histological subtype. Adjuvant hysterectomy was performed between three and twelve weeks after chemoradiation therapy. Residual disease in the pathology specimen was found in 34% of the patients. All studies performed adjuvant hysterectomy at different time points after chemoradiation therapy. No association was found for pathology-proven residual disease regarding the time between chemoradiation therapy and adjuvant hysterectomy (Figure 2).

Recurrence of disease after treatment with chemoradiation therapy and adjuvant hysterectomy was observed in 270 patients (22%). Recurrence was pathology-proven in the study of Sun et al. In all other studies the way of determining the recurrence of disease is unknown. The survival rate is based on ten studies and 1115 patients, of which 298 patients (27%) deceased because of cervical cancer and 28 patients (3%) deceased because of causes unrelated to cervical cancer. The follow-up period is described in ten studies and the patients were followed during a range of 2.4 and 245 months (Table 1).

A total of 202 complications related to adjuvant hysterectomy were registered in 578 patients treated with chemoradiation therapy and adjuvant hysterectomy (35%). A total of 27 fistulas (5%) was reported, of which sixteen fistulas to the urinary tract and eleven fistulas to the gastro-intestinal tract. A total of 53 complications of the urinary tract was reported (9%) five injuries to the urinary tract and five ureteral stenoses. Six complications to the female genital tract were reported (1%), two patients with vaginal necrosis and two with vaginal stenosis. Eighteen gastro-intestinal complications were reported (3%) of which two intestinal injuries and a total of thirteen infections was reported (2%) of which seven with an abscess, four with peritonitis and two patients with pelvic infections. Other complications were reporter 81 times, of which four deaths related to postoperative morbidity, four ruptures of the iliac vessel during surgery and one pulmonary embolism (Table 3).

## 4. Discussion

This systematic review shows a mean pathology-proven residual disease in 34% (range, 14–52%) of all patients undergoing adjuvant hysterectomy after chemoradiation therapy for cervical cancer. No association was found between the time between chemoradiation therapy and for pathology-proven residual disease on adjuvant hysterectomy specimens. The survival rate after chemoradiation therapy and adjuvant hysterectomy seem suboptimal, and adjuvant hysterectomy has a high risk of complications, including death. 

### 4.1. Comparison with Existing Literature

In this systematic review, the percentage of residual disease on adjuvant hysterectomy specimens did not diminish in time. In the included studies, adjuvant hysterectomy was performed within twelve weeks after chemoradiation therapy. However, we believe it might be plausible that the incidence of positive pathology could decrease because the morphologic response sometimes continues for several weeks after the last treatment of chemoradiation therapy [10]. In addition to this hypothesis, Eifel et al. reported 22% local recurrences for patients with stage III cervical cancer after treatment with only chemoradiation therapy [5]. Which is lower than the mean 34% residual disease in the surgical specimen in our review. This may indicate that when (salvage) surgery is performed too early after chemoradiation therapy patients are overtreated. Thereby, two previously published studies assessed residual disease detection by biopsy. The study of Boers et al. shows that residual disease detected by biopsy eight to ten weeks after chemoradiation therapy is a poor prognostic factor to identify patients with residual disease who may be salvaged by surgery [24]. The study by Hoeijmakers et al. implies that a biopsy to prove residual disease should be taken twelve until sixteen weeks after completing chemoradiation therapy, to select patients for salvage surgery [25]. These studies indicate that detection of residual disease should not take place early after chemoradiation therapy. 

As previously mentioned, two recent meta-analyses determined the survival rates for adjuvant hysterectomy after chemoradiation. They concluded that adjuvant hysterectomy seemed to reduce the risk of local recurrence [8,9]. One limitation of these studies is that the results are based on only two randomized controlled trials [26,27]. The review of literature in our study shows an overall recurrence rate (distant and local recurrence) of 22% after adjuvant hysterectomy and chemoradiation therapy. Previous studies reported an overall recurrence rate after treatment with only chemoradiation therapy of up to 37% [5,6]. We reported an overall survival of 73% after chemoradiation therapy and adjuvant hysterectomy, which seems to be equivalent to treatment with chemoradiation therapy alone. Previous studies reported 5-year overall survival rates for patients with stage IB-IVA ranges between 32% and 83% [7]. A hypothesis for the decreased recurrence rate after adjuvant hysterectomy and chemoradiation therapy, compared to chemoradiation therapy alone, is that patients with residual disease after chemoradiation therapy are directly treated with salvage surgery. Patients with residual disease after chemoradiation therapy are an unfavorable group of patients, which are likely to relapse. However, standard adjuvant hysterectomy after chemoradiation therapy harbors the risk that the majority of patients are unnecessary treated and thus exposed to the (post)operative risks. Besides, more recent studies use a higher total dose of radiation therapy, which results in lower reported local recurrence rates. For example, the retroEMBRACE study used a mean D90 (EQD210) for high and intermediate risk clinical target volumes of 87 ± 15 Gy and 69 ± 8 Gy respectively. They reported 9.6% of local failures, of which 6.4% had true local recurrences [7]. This indicates even less reason to perform an adjuvant hysterectomy. 

Additionally, adjuvant hysterectomy after chemoradiation therapy is associated with a high complication risk. The risk of complications after adjuvant hysterectomy is higher in comparison with primary surgery because adjuvant hysterectomy takes place in a previously irradiated area with the unpredictable healing quality of the tissue [28]. In our systematic review adjuvant hysterectomy is associated with a percentage of major complications of 35%. The mortality rate after adjuvant hysterectomy in our systematic review is 0.7%. The study of Magrina et al. reported a mortality rate of 0.5% after primary radical hysterectomy [29]. A major complication with high implications for patients is the development of a fistula. Fistulas can occur due to irradiation, surgery, or a combination of both [30]. The study of Hilton et al. reported an overall fistula rate after a hysterectomy of 0.13% and a fistula rate of 1% after radical hysterectomy for patients with cervical cancer [31]. In this review the studies reported an overall fistula rate of 5%, this implicates that the chance of developing fistulas increases after the combination of chemoradiation therapy and adjuvant hysterectomy. Most of the complications after adjuvant hysterectomy turn out to be unnecessary because 66% of the patients were overtreated with adjuvant hysterectomy. Patients should be clearly informed on the survival benefits and the disadvantages like complications and the over-treatment rate of non-routinely adjuvant hysterectomy. To reduce the number of patients needed to harm by adjuvant hysterectomy, less invasive procedures, such as radiological imaging or biopsies, should be performed first, to determine the presence of residual disease before performing surgery. However, the best period to determine residual disease by radiological imaging, biopsy, as well as performing adjuvant or salvage surgery, should be further investigated. 

### 4.2. Strengths and Weaknesses

One of the strengths of this study is that the dose and regimen of chemoradiation therapy that patients received were assessed by a radiation oncologist, this ensures that all patients were treated via a comparable chemoradiation therapy treatment schedule, similar to high income countries. However, the evaluation of data is not without limitations, there is much heterogenicity among the included studies. First, the time to complete chemoradiation therapy was not an inclusion criterion, which could influence survival outcomes. Second, not all studies performed the same type of adjuvant hysterectomy after chemoradiation therapy. Differing between simple hysterectomy and extended hysterectomy with or without lymph node dissection, and between open versus laparoscopic surgery. This could influence the complication rate. Analyzing the different surgery types regarding recurrence, survival and complications was not possible because of missing data. Third, the preoperative doses of chemoradiation therapy given to patients were nearly always less than the planned dose. Fourth, pathological residual disease rate can be underestimated because the viable microscopic residual disease can be missed in surgical specimens. Fifth, it was not possible to calculate the 2-, 3- or 5-years overall survival rate as the data was not representative, which could overestimate or underestimate the survival rate.

## 5. Conclusions

The majority of patients treated with adjuvant hysterectomy as a routine procedure are over-treated. No association was found between the time between chemoradiation therapy and pathology-proven residual disease on adjuvant hysterectomy specimens. The survival rate after adjuvant hysterectomy seems suboptimal, and the risk of major complications after adjuvant hysterectomy is high. To prevent high overtreatment rates by adjuvant hysterectomy it should be considered to use less invasive procedures to determine the presence of residual disease before performing surgery after chemoradiation therapy. Patients need to be well informed on potential complications, survival benefits, and a potential overtreatment rate before considering an adjuvant hysterectomy.

## Figures and Tables

**Table 1:** Characteristics of included studies. Patients treated with chemoradiation therapy including brachytherapy and adjuvant hysterectomy. Studies are shown based on time until adjuvant hysterectomy.

Author (Year)	Inclusion Period (Years)	Total Number of Patients (Treated with Chemoradiation Therapy and Adjuvant Hysterectomy)	Age Median/Mean (Range)	FIGO Stage *^1^	Histology	Chemoradiation Therapy regimen	Brachytherapy Dosage	Type of Adjuvant Hysterectomy	Time Until Adjuvant Hysterectomy	Positive Pathology Results After Surgery *n* (%)	Follow-up Period Median/Mean (Range)	Recurrence *n* (%)	Survival *n* (%)
Keys (1999) [13]	1992–1997	374 (183)	Unknown	IB = 183	SCC = 147, AC = 9, ASC = 17, Other = 10	45 Gy EBRT with concomitant cisplatin (40 mg/m^2^) *^2^	30 Gy	Extrafascial hysterectomy *^3^	3–6 weeks	88 (48%)	Median 26 months	38 (21%)	27 died (15%)
Eitan (2010) [14]	2003–2006	23	Median 50 year (range, 30–67 year)	IB2 = 20, IIA = 3	SCC = 22, AC = 1	45 Gy EBRT with concomitant cisplatin (35 mg/m^2^) *^4^	14 Gy (2 × 7 Gy)	Simple extra-fascial total abdominal hysterectomy and bilateral salpingo-oophorectomy (and in 16 patients PLND).	Median 5 weeks, range 14–52 days.	11 (48%)	Median 20 months (range, 10–50 months)	4 (17.4%)	2 (8.7%)
Colombo (2009) [15]	2000–2008	102	Mean 44 year (range, 24–74 year)	IB = 28, IIA = 13, IIB = 61	SCC = 91 AC = 10 other = 1	45 Gy EBRT with concomitant cisplatin (40 mg/m^2^.	15 Gy *^5^	56 abdominal radical hysterectomy and 46 total laparoscopic radical hysterectomy	4–6 weeks	36 (35%)	Mean 31.2 months	32 (31.4%): 18 (17.6%) local and 14 (13.7%) distant	3-year OS 82%, 19 died (18.6%)
Fröbe (2014) [16]	2002–2008	24	Median 50 year (range, 39–71 year)	IB = 8, IIA = 3, IIB = 13	SCC = 19 AC = 5	40 Gy EBRT with concomitant cisplatin (30 mg/m^2^) *^6^	28 Gy (4 × 7 Gy)	Radical hysterectomy and bilateral salphingo-oophorectomy without lymph node dissection	6 weeks	12 (50%)	Median 67 months (range 4–107 months)	three distant metastases (12.5%)	Six died (25%) three without evidence of disease
Cheewakriangkrai (2005) [17]	1999–2001	34	Mean 44 year (range, 30–66 year)	IB1 = 4, IB2 = 25, IIA = 5	SCC = 22 AC = 11 ASC = 1	46–50 Gy ERBT with weekly concomitant cisplatin (40 mg/m^2^).	30 Gy (4 × 7.5 Gy)	Extra-fascial hysterectomy	6 weeks	16 (47%)	Median 42 months (range 7–58 months)	Six (18.2%):one distant, three local and two combined	five deaths (14.7%)
Cagetti (2020) [18]	2012–2017	145 (90)	Median 54 year (range, 24–90 year)	IB = 33, IIA = 9, IIB = 40, III-IV = 8	SCC = 66, AC = 19, other = 5	45 Gy EBRT with concomitant cisplatin (40 mg/m^2^) *^7^	27.5 Gy (5 × 5.5 Gy)	Radical hysterectomy	5–8 weeks median: 61 ± 26 days	28 (31%)	Median 30.8 months (range, 26.0–30.0 months)	11 local (12.2%)	3-year OS 50–90% depending on pathology results
Bigsby (2011) [19]	1999–2009	69	Mean 47 year (range, 27–82 year)	IB2 = 69	SCC = 55 AC = 11 ASC = 3	45–50.4 Gy EBRT with concomitant cisplatin (25–40 mg/ m^2^) *^8^	15–18 Gy (3 × 5–6 Gy)	Extra-fascial total abdominal hysterectomy with common and para-aortic lymphadenectomy	6–8 weeks	35 (51%)	Mean 61.7 months (range 10.9–122.5 months)	16 (23.2%) 2 local, 14 distant	16 deaths (23.2%) three were of unrelated causes.
Kunos (2010) [20]	Unknown	464 (175)	Median 40 year (range, 21–81 year)	IB = 175	SCC = 140, AC = 9, ACS = 16, Other = 10	45 Gy EBRT with concomitant cisplatin (40 mg/m^2^)	30 Gy	Total extrafascial. hysterectomy	6–8 weeks	35 (20%) *^9^	Median 128 months	39 (22.3%): 16 (9%) local, 23 (13%) distant	30 cancer related deaths, 15 death of unrelated cause.
Zheng (2017) [21]	2008–2013	314 (163)	Median 51 (range, 26–73 year)	IB2 = 35, IIA = 71, IIB = 57	SCC = 136, AC = 27	46–50 Gy EBRT with concomitant cisplatin (40 mg/m^2^) *^10^	25–30 Gy	Radical hysterectomy and PLND	Within 8 weeks	65 (39.9%)	Unknown	48 (29.4%) 12 local, 29 metastases, seven local and distant	3-year OS 87.1% 21 deaths (12.9%)
Touboul (2009) [22]	1998–2007	150	Median 47 year (range, 19–77 year)	IB2 = 48, II = 91, III = 10, IV = 1	SCC = 108 AC = 26 other = 16	45 Gy EBRT with concomitant cisplatin (40 mg/m^2^) *^11^	15 Gy	Radical hysterectomy (*n* = 44) or simple extra fascial hysterectomy (*n* = 106) with or without para-aortic and/or PLND	8–10 weeks	78 (52%)	Median 43.2 months (range, 2.4–127.2 months)	41 (27%)	37 deaths (24.7%)
Sun (2013) [23]	1992–2012	378 (192)	Median 48 year (range, 20–75 year)	IIB = 90, III = 101, IVA = 1	SCC = 149 AC= 30 other= 13	44–55 Gy EBRT with concurrent cisplatin (40 mg/m^2^) and 5-FU (500 mg/m^2^)	45–55 Gy	99 Extra-fascial hysterectomy and 81 Extended hysterectomy 12 other	10–12 weeks	27 (14%)	Median 190 months (range 60–245)	32 (16.7%)	60 deaths (31.1%)
Total		2077(1205)	Range, 19–90 years	IB = 427, IB1 = 4, IB2 = 197, II = 91, IIA = 104, IIB = 261, III = 111, IV = 1 IVA = 1 III-IVA = 8	SCC = 955, AC = 158, ASC = 37, Other = 55					411(34%)	Range 2.4–245 months	270 local/distant	223 died based on 10 studies of which 21 died of unrelated causes to cervical cancer.

CRT = chemoradiation therapy. FIGO = International Federation of Gynecology and Obstetrics. SCC= squamous cell carcinoma. AC = adenocarcinoma. ASC = adenosquamous carcinoma. EBRT = external beam radiation therapy. Gy = Gray. 5-FU= 5-fluoruracl. PLND= pelvic lymph node dissection. OS= overall survival. *^1^ All patients were staged before the FIGO 2018 staging system. *^2^ Four patients (2%) were allocated to CRT group and received cisplatin. 90% received four or more courses of cisplatin. Median dose cisplatin 39 mg/m^2^. Two patients (1%) refused to undergo radiotherapy. *^3^ Eight patients (4%) did not undergo adjuvant hysterectomy. *^4^ All but two patients (9%) received the full planned dose EBRT. One patient received one additional dose of 1.8 Gy, and one patient missed one dose of 1.8 Gy. Six patients received five cycles of cisplatin instead of six (26%) and one patient (4%) received four cycles of cisplatin. *^5^ Six patients (6%) did not receive brachytherapy. *^6^ Sixteen patients (67%) received 40 Gy in 22 fractions, four patients (17%) received between 42–46 Gy in 23–25 fractions and two patients (8%) received 50.4 Gy in 28 fractions. Two patients (8%) received doses under 40 Gy (28.8 Gy and 37.8 Gy), two patients (8%) had only two cycles of cisplatin. *^7^ Median dose 45 Gy (range 43.2–50.4 Gy). 35 patients (39%) received a parametrial boost EBRT. Cisplatin was given in 94.9%, in case of contra-indication carboplatin was delivered (5.1%). *^8^ 9% did not received the recommended dose of EBRT, three patients received lower dose (41.4 Gy) and three a higher dose (54.0 Gy). Brachytherapy was omitted in four patients (6%) because of upper vaginal stenosis and 23 (35%) received brachytherapy outside of the protocol guidelines: 14 patients (8%) received less brachytherapy (5–12 Gy) and nine patients (5%) received more brachytherapy (20–30 Gy). 49 patients (71%) received cisplatin 40 mg/m^2^, 17 patients (25%) received 25 mg/m^2^ and three patients (4%) received cisplatin/5FU. *^9^ Positive pathology is defined when equal or more than 10% tumor cells are viable in de specimen. *^10^ Thirteen patients (8%) were treated with four cycles, 31 patients (19%) with five cycles and 119 patients (73%) with six cycles cisplatin. *^11^ Patients with parametrial spread and/or bulky pelvic nodes on imaging received a pelvic lateral boost of 10–15 Gy, unknown how much patients received this boost.

**Table 2:** Newcastle Ottawa scale for cohort studies.

Article	Selection				Comparability	Outcome			Quality
	Representativeness of the Exposed Cohort *	Selection Cohorts’ Same Source	Ascertainment of Exposure **	Outcome of Interest was not Present at Start of Study	Comparability of Cohorts	Assessment of Outcome ***	Follow-Up ****	Adequacy of Follow-Up	
Keys (1999) [13]	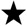	NA	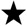	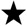	NA	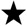	Median 36 months	No statement about lost to follow-up	Fair
Etian (2010) [14]	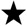	NA	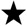	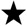	NA	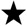	Median 20 months (range 10–50 months) 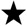	No statement about lost to follow-up	Fair
Colombo (2009) [15]	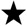	NA	Not reported	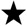	NA	Not reported	Median 31 months	No statement about lost to follow-up	Poor
Fröbe (2014) [16]	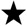	NA	Not reported	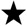	NA	Not reported	Median 5.59 years (range 0.32–8.9 year) 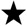	No patient lost to follow-up 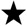	Fair
Chweewakriangkrai (2005) [17]	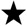	NA	Not reported	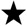	NA	Not reported	Median 42 months (range 7–58 months) 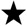	1 patient was lost to follow-up 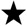	Fair
Cagetti (2020) [18]	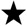	NA	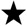	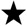	NA	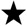	Median 30.8 months (range, 26.0- 30.0 months) 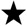	No statement about lost to follow-up	Fair
Bigsby (2010) [19]	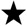	NA	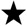	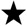	NA	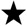	Mean 71.7 months (range 10.9–122.5 months) 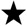	No patients lost to follow-up 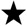	Good
Kunos (2010) [20]	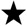	NA	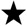	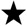	NA	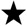	Median 118 months	Three patients lost to follow-up 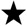	Fair
Zheng (2017) [21]	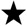	NA	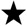	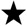	NA	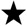	No follow-up period reported	No statement about lost to follow-up	Fair
Touboul (2014) [22]	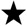	NA	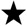	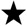	NA	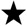	Median 3.6 years (range 0.2–10.6 year) 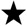	Four patients lost to follow up 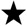	Good
Sun (2013) [23]	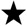	NA	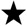	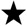	NA	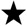	Median 190 months (range 60–245 months) 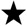	No statement about lost to follow-up	Fair

* Representativeness of the exposed cohort: all included studies representative for women with residual cervical cancer after chemoradiation therapy treated with salvage surgery. ** Ascertainment of exposure: all with database or medical records. *** Assessment of outcome: all with medical records. **** Follow-up period ≥ 12 months was assessed as long enough for outcomes to occur. 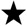: fulfilled the criteria of the Newcastle Ottawa Scale for this item.

**Table 3:** Complications after chemoradiation therapy including brachytherapy followed by adjuvant hysterectomy.

Auteur (Year)	Patients Treated with Adjuvant Hysterectomy	Fistula	Urinary Tract	Female Genital Tract	Gastrointestinal	Infection	Other	Total
Eitan (2010) [14]	23	One recto-vaginal fistula	10 cystitis		11 diarrheas		Seven anemia, two lymphedema	31
Colombo (2009) [15]	144	Seven urinary fistulas, two digestive fistulas	15 vesical dysfunction, 12 cystitis, three bladder injury, two ureteral injury		Two digestive injury	Two pelvic infections	Six intraoperative hemorrhages, five other, two symptomatic lymphocysts, one pulmonary embolism, one postoperative hemorrhage	64
Bigsby (2011) [19]	69	One enterocutaneous fistula, one rectovaginal fistula	One ureteral stenosis requiring stent, one cystotomy with repair	Two vaginal stenosis, one vaginal fault necrosis/grade four proctitis, one vaginal vault necrosis,	one complete small bowel obstruction		One deep venous thrombosis	10
Touboul (2009) [22]	150	Five ureteral fistula, five bowel fistulas, one bladder fistula	Two ureteral stenosis, two bladder retention, one urinary incontinence	One vaginal vault dehiscence with abscess	Two bowel obstruction, one epigastralgia	Five abscesses, three peritonitis	Nine lymphedemas, eight lymphocysts, three chylous ascites, three phlebitis, two deaths related to postoperative morbidity, two rupture of iliac vessels, two wound dehiscence	57
Sun (2013) [23]	192	Two ureteral fistula, one bowel fistula, one bladder fistula	Two ureteral stenosis, one bladder retention, one urinary incontinence	One vaginal vault dehiscence with abscess	One bowel obstruction	Two abscesses, one peritonitis	15 lymphocysts, three lymphedemas, two death related to postoperative morbidity, two chylous ascites, two ruptures of iliac vessels, two wound dehiscence, one phlebitis	40
Total	578	27	53	6	18	13	81	202

## Supplementary Materials

The following are available online at www.mdpi.com/article/10.3390/cancers13246190/s1, supporting information S1: Literature search

## References

[1] WHO Cervical Cancer 2019. https://www.who.int/cancer/prevention/diagnosis-screening/cervical-cancer/en/.

[2] Bhatla N., Aoki D., Sharma D.N., Sankaranarayanan R. (2018). Cancer of the cervix uteri. Int. J. Gynecol. Obstet..

[3] Cancer Research UK Cancer Statistics for the UK. https://www.cancerresearchuk.org/health-professional/cancer-statistics-for-the-uk.

[4] Cibula D., Pötter R., Planchamp F., Avall-Lundqvist E., Fischerova D., Haie Meder C., Köhler C., Landoni F., Lax S., Lindegaard J.C. (2018). The European Society of Gynaecological Oncology/European Society for Radiotherapy and Oncology/European Society of Pathology Guidelines for the Management of Patients With Cervical Cancer. Int. J. Gynecol. Cancer.

[5] Eifel P.J., Winter K., Morris M., Levenback C., Grigsby P.W., Cooper J., Rotman M., Gershenson D., Mutch D.G. (2004). Pelvic irradiation with concurrent chemotherapy versus pelvic and para-aortic irradiation for high-risk cervical cancer: An update of Radiation Therapy Oncology Group Trial (RTOG) 90-01. J. Clin. Oncol..

[6] Pötter R., Dimopoulos J., Georg P., Lang S., Waldhäusl C., Wachter-Gerstner N., Weitmann H., Reinthaller A., Knocke T.H., Wachter S.K.C. (2007). Clinical impact of MRI assisted dose volume adaptation and dose escalation in brachytherapy of locally advanced cervix cancer. Radiother. Oncol..

[7] Sturdza A., Pötter R., Fokdal L.U., Haie-Meder C., Tan L.T., Mazeron R., Petric P., Šegedin B., Jurgenliemk-Schulz I.M., Nomden C. (2016). Image guided brachytherapy in locally advanced cervical cancer: Improved pelvic control and survival in RetroEMBRACE, a multicenter cohort study. Radiother. Oncol..

[8] Shi D., Liang Z., Zhang C., Zhang H.L.X. (2018). The effect of surgery on the survival status of patients with locally advanced cervical cancer after radiotherapy/chemoradiotherapy: A meta-analysis. BMC Cancer.

[9] Shim S.H., Kim S.N., Chae S.H., Kim J.E.L.S. (2018). Impact of adjuvant hysterectomy on prognosis in patients with locally advanced cervical cancer treated with concurrent chemoradiotherapy: A meta-analysis. J. Gynecol. Oncol..

[10] Baskar R., Lee K.A., Yeo R., Yeoh K.W. (2012). Cancer and radiation therapy: Current advances and future directions. Int. J. Med. Sci..

[11] Kol K.G.G., Ebisch R.M.F., Piek J.M.J., Zusterzeel P.L.M., Vergeldt T.F.M., Bekkers R.L.M. (2021). Salvage surgery for patients with residual disease after chemoradiation therapy for locally advanced cervical cancer: A systematic review on indication, complications, and survival. Acta Obs. Gynecol. Scand..

[12] Viswanathan A.N., Lee L.J., Eswara J.R., Horowitz N.S., Konstantinopoulos P.A., Mirabeau-Beale K.L., Rose B.S., Von Keudell A.G., Wo J.Y. (2014). Complications of pelvic radiation in patients treated for gynecologic malignancies. Cancer.

[13] Keys H.M., Bundy B.N., Stehman F.B., Muderspach L.I., Chafe W.E., Suggs C.L., Walker J.L., Gersell D., Mackey D. (1999). Cisplatin, radiation, and adjuvant hysterectomy compared with radiation and adjuvant hysterectomy for bulky stage IB cervical carcinoma. N. Engl. J. Med..

[14] Eitan R., Levavi H., Peled Y., Brenner R., Sabah G., Ben-Arie A., Dgani R., Fishman A., Sulkes A., Fenig E. (2010). Should simple hysterectomy be added after chemo-radiation for stage IB2 and bulky IIA cervical carcinoma?. Aust. N. Z. J. Obs. Gynaecol..

[15] Colombo P.E., Bertrand M.M., Gutowski M., Mourregot A., Fabbro M., Saint-Aubert B., Quenet F., Gourgou S., Kerr C., Rouanet P. (2009). Total laparoscopic radical hysterectomy for locally advanced cervical carcinoma (stages IIB, IIA and bulky stages IB) after concurrent chemoradiation therapy: Surgical morbidity and oncological results. Gynecol. Oncol..

[16] Fröbe A., Jones G., Bokulić T., Mrčela I., Budanec M., Murgić J., Jakšić B., Prpić M., Bolanča A., Kusić Z. (2014). High-dose-rate brachytherapy and concurrent chemoradiotherapy followed by surgery for stage Ib-IIb cervical cancer: Single institution experience. Anticancer Res..

[17] Cheewakriangkrai C., Srisomboon J., Chitapanarux I., Suprasert P., Phongnarisorn C., Sitthicha Siriaree K.C. (2005). Concurrent cisplatin-based chemoradiation and adjuvant hysterectomy for bulky stage IB-IIA cervical cancer. J. Med. Assoc. Thai..

[18] Cagetti L., Zemmour C., Minsat M., Lambaudie E., Houvenaeghel G., Provansal M., Cappiello M.A., Rua S., Jauffret C., Ferré M. (2020). Lessons from radiochemotherapy and modern image-guided adaptive brachytherapy followed by hysterectomy. Gynecol. Oncol..

[19] Bigsby G.E., Robert W., Holloway Ahmad S., Michael D., Sombeck G.E. (2012). Chemoradiation with adjuvant hysterectomy for stage IB-2 cervical cancer: A 10-year experience. Gynecol. Surg..

[20] Kunos C., Ali S., Abdul-Karim F.W., Stehman F.B., Waggoner S. (2010). Posttherapy residual disease associates with long-term survival after chemoradiation for bulky stage 1B cervical carcinoma: A Gynecologic Oncology Group study. Am. J. Obs. Gynecol..

[21] Zheng D., Mou H.P., Diao P., Li X.M., Zhang C.L., Jiang J., Chen J.L., Wang L.S., Wang Q., Zhou G.Y. (2018). Chemoradiotherapy in combination with radical surgery is associated with better outcome in cervical cancer patients. Oncotarget.

[22] Touboul C., Uzan C., Mauguen A., Gouy S., Rey A., Pautier P., Lhommé C., Duvillard P., Haie-Meder C., Morice P. (2010). Prognostic Factors and Morbidities After Completion Surgery in Patients Undergoing Initial Chemoradiation Therapy for Locally Advanced Cervical Cancer. Oncologist.

[23] Sun L., Sheng X., Jiang J., Li X., Liu N., Liu Y., Zhang T., Li D., Zhang X., Wei P. (2014). Surgical morbidity and oncologic results after concurrent chemoradiation therapy for advanced cervical cancer. Int. J. Gynecol. Obstet..

[24] Boers A., Arts H.J.G., Klip H., Nijhuis E.R., Pras E., Hollema H., Wisman G.B.A., Nijman H.W., Mourits M.J.E., Reyners A.K.L. (2014). Radical surgery in patients with residual disease after (chemo)radiation for cervical cancer. Int. J. Gynecol. Cancer.

[25] Hoeijmakers Y.M., Snyers A., van Ham M.A.P.C., Zusterzeel P.L.M., Bekkers R.L.M. (2019). Cervical biopsy after chemoradiation for locally advanced cervical cancer to identify residual disease: A retrospective cohort study. J. Surg. Oncol..

[26] Morice P., Rouanet P., Rey A., Romestaing P., Houvenaeghel G., Boulanger J.C., Leveque J., Cowen D., Mathevet P., Malhaire J.P. (2012). NoResults of the GYNECO 02 study, an FNCLCC phase III trial comparing hysterectomy with no hysterectomy in patients with a (clinical and radiological) complete response after chemoradiation therapy for stage IB2 or II cervical cancer. Oncologist.

[27] Cetina L., Garcia-Arias A., Candelaria M., Cantú D., Rivera L., Coronel J., Bazan-Perkins B., Flores V., Gonzalez A.D.G.A. (2009). Brachytherapy versus radical hysterectomy after external beam chemoradiation: A non-randomized matched comparison in IB2-IIB cervical cancer patients. World J. Surg. Oncol..

[28] Wydra D., Emerich J., Sawicki S., Ciach K., Marciniak A. (2006). Major complications following exenteration in cases of pelvic malignancy: A 10-year experience. World J. Gastroenterol..

[29] Magrina J.F., Goodrich M.A., Weaver A.L., Podratz K.C. (1995). Modified radical hysterectomy: Morbidity and mortality. Gynecol. Oncol..

[30] Narayanan P., Nobbenhuis M., Reynolds K.M., Sahdev A., Reznek R.H., Rockall A.G. (2009). Fistulas in malignant gynecologic disease: Etiology, imaging, and management. Radiographics.

[31] Hilton P., Cromwell D.A. (2012). The risk of vesicovaginal and urethrovaginal fistula after hysterectomy performed in the English National Health Service-a retrospective cohort study examining patterns of care between 2000 and 2008. BJOG An. Int. J. Obs. Gynaecol..

