# Supplementary material for: Adjuvant Hysterectomy for Cervical Cancer Patients Treated with Chemoradiation Therapy: A Systematic Review on the Pathology-Proven Residual Disease Rate
Source: Cancers (Basel). 2021 Dec 8;13(24):6190. doi: 10.3390/cancers13246190 (PMC8699574; doi:10.3390/cancers13246190)
Supplement: Supplementary file 1 [file cancers-13-06190-s001.zip › cancers-1475808-supplementary.pdf]

S1 literature search
Search pubmed
08 March 2021

((((((("Cervix Uteri"[Mesh])) OR ("Cervix Uteri"[tiab] OR Cervix*[tiab] OR "Uterine Cervix"[tiab] OR Cervix[tiab] OR Cervical[tiab] OR "cervical cancer"[tiab])) OR ("Uterine Cervical Neoplasms"[Mesh])) OR (Uterine Cervical Neoplasm*[tiab] OR Cervical Neoplasm*[tiab] OR Cervix Neoplasm*[tiab] OR "Cancer of the Uterine Cervix"[tiab] OR "Cancer of Cervix"[tiab]))) AND ((((("Chemoradiotherapy"[Mesh])) OR ("Chemoradiotherapy"[tiab] OR Chemoradiotherapies[tiab] OR Radiochemotherapy[tiab] OR Radiochemotherapies[tiab])) OR (chemoradiation[tiab])) OR (radiation[tiab]))) AND (((("Hysterectomy"[Mesh])) OR (Hysterectomy[tiab] OR hysterectomies[tiab])) OR ("adjuvant surgery"[tiab]))

**MeSH**	**tiab**
Cervix uteri	Cervix uteri
	Cervix
	Uterine Cervix
	Cervix
	Cervical
	Cervical cancer
Uterine Cervical Neoplasms	Uterine Cervical Neoplasm*
	Cervical Neoplasm*
	Cervix Neoplasm*
	Cancer of the Uterine Cervix
	Cancer of Cervix
Chemoradiotherapy	Chemoradiotherapy
	Chemoradiotherapies
	Radiochemotherapy
	Radiochemotherapies
	Chemoradiation
	Radiation
Hysterectomy	Hysterectomy
	Hysterectomies
	Adjuvant surgery

Search EMBASE

**Exp**	**tiab**
uterine cervix tumor/	Cervix uteri.ti. or Cervix uteri.ab.
exp uterine cervix/	Cervix.ti. or Cervix.ab.
	Uterine Cervix.ti. or Uterine Cervix.ab.
	Cervical.ti. or Cervical.ab.
	Cervical cancer.ti. or Cervical cancer.ab.
	Uterine Cervical Neoplasm*.ti. or Uterine Cervical Neoplasm*.ab.
	Cervical Neoplasm*.ti. or Cervical Neoplasm*.ab.
	Cancer of the Uterine Cervix.ti. or Cancer of the Uterine Cervix.ab.
	Cancer of Cervix.ti. or Cancer of Cervix.ab.
	Cervix Neoplasm*.ti. or Cervix Neoplasm*.ab.

exp chemoradiotherapy/	Chemoradiotherapy.ti. or Chemoradiotherapy.ab.
	Chemoradiotherapies.ti. or Chemoradiotherapies.ab.
	Radiochemotherapy.ti. or Radiochemotherapy.ab.
	Radiochemotherapies.ti. or Radiochemotherapies.ab.
	chemoradiation.ti. or chemoradiation.ab.
	radiation.ti. or radiation.ab.
exp hysterectomy/	hysterectomy.ti. or hysterectomy.ab.
	Hysterectomies.ti. or Hysterectomies.ab.
	Adjuvant surgery.ti. or Adjuvant surgery.ab.

Search Cochrane

**MeSH**	**Ti,ab,kw**
Cervix uteri	Cervix
	Uterine cervical cancer
	Uterine cervix
	Cervical
	Cervical cancer
Uterine cervical neoplasms	
Chemoradiotherapy	Chemoradiotherapy
	Chemoradiotherapies
	Radiochemotherapy
	Chemoradiation
Hysterectomy	Hysterectomy
	Hysterectomies
	Adjuvant surgery
